# Proteomic profiling of skeletal and cardiac muscle in cancer cachexia: alterations in sarcomeric and mitochondrial protein expression

**DOI:** 10.18632/oncotarget.25146

**Published:** 2018-04-24

**Authors:** Angie M. Y. Shum, Anne Poljak, Nicholas L. Bentley, Nigel Turner, Timothy C. Tan, Patsie Polly

**Affiliations:** ^1^ Mechanisms of Disease and Translational Research, School of Medical Sciences, Faculty of Medicine, UNSW Sydney, New South Wales, Australia; ^2^ Department of Pathology, School of Medical Sciences, Faculty of Medicine, UNSW Sydney, New South Wales, Australia; ^3^ Department of Pharmacology, School of Medical Sciences, Faculty of Medicine, UNSW Sydney, New South Wales, Australia; ^4^ Bioanalytical Mass Spectrometry Facility, UNSW Sydney, New South Wales, Australia; ^5^ Centre for Healthy Brain Ageing, School of Psychiatry, UNSW Sydney, New South Wales, Australia; ^6^ Western Clinical School and Westmead Hospital, Westmead, New South Wales, Australia

**Keywords:** cancer cachexia, muscle, skeletal, cardiac, proteomics

## Abstract

**Background:**

Cancer cachexia is observed in more than 50% of advanced cancer patients, and impairs quality of life and prognosis. A variety of pathways are likely to be dysregulated. Hence, a broad-spectrum understanding of the disease process is best achieved by a discovery based approach such as proteomics.

**Results:**

More than 300 proteins were identified with > 95% confidence in correct sequence identification, of which 5–10% were significantly differentially expressed in cachectic tissues (*p*-value of 0.05; 27 proteins from gastrocnemius, 34 proteins from soleus and 24 proteins from heart). The two most pronounced functional groups being sarcomeric proteins (mostly upregulated across all three muscle types) and energy/metabolism proteins (mostly downregulated across all muscle types). Electron microscopy revealed disintegration of the sarcomere and morphological aberrations of mitochondria in the cardiac muscle of colon 26 (C26) carcinoma mice.

**Materials and Methods:**

The colon 26 (C26) carcinoma mouse model of cachexia was used to analyse soleus, gastrocnemius and cardiac muscles using two 8-plex iTRAQ proteomic experiments and tandem mass spectrometry (LCMSMS). Differentially expressed proteomic lists for protein clustering and enrichment of biological processes, molecular pathways, and disease related pathways were analysed using bioinformatics. Cardiac muscle ultrastructure was explored by electron microscopy.

**Conclusions:**

Morphological and proteomic analyses suggested molecular events associated with disintegrated sarcomeric structure with increased dissolution of Z-disc and M-line proteins. Altered mitochondrial morphology, in combination with the reduced expression of proteins regulating substrate and energy metabolism, suggest that muscle cells are likely to be undergoing a state of energy crisis which ultimately results in cancer-induced cachexia.

## INTRODUCTION

Tumours not only interrupt local tissue function but have been shown to produce an additional systemic effect on the entire metabolism of the body. A current paradigm of cancer pathology has inflammatory processes as central to many of the paraneoplastic symptoms observed in cancer patients, such as cancer cachexia, which is observed in more than 50% of advanced cancer patients [1, 2]. It is characterised by progressive weight loss associated with skeletal muscle atrophy and depletion of adipose tissue, irrespective of nutritional intake [3]. Lower performance status and global quality of life with higher fatigue scores and pain have been observed in patients who lost weight with gastrointestinal cancer or non-small cell lung cancer [4, 5]. At least 20% of deaths are caused by cachexia in part due to impaired respiratory or cardiac function [6, 7]. Moreover, the loss of body weight has been observed in a number of patients with various cancers with median survival of less than 6 months, and hence is an indicator of terminal disease [8]. Weight loss has also been associated with poor prognosis, higher recurrence and worse response to chemotherapy [9–11]. Importantly, it has been shown that the attenuation of weight loss would significantly improve overall survival in patients with gastrointestinal cancer [12]. Taken together, cancer cachexia significantly impairs cancer patient quality of life and prognosis, and reversal of this condition would be beneficial to the management of malignancy in general.

An effective therapeutic option is currently not available to manage cancer cachexia. This is partly due to a limited understanding of the molecular mechanisms underlying this disease process, highlighting the need to better define mediators and factors implicated in the pathology. Research in the field has largely been driven by candidate gene approaches in which specific molecules are selected for study based on their cellular roles. However, it is becoming apparent that cancer cachexia is a complex multi-factorial disease which may involve multiple pathways, and candidate molecular approaches may therefore have limited value in providing insights for overall molecular interactions which are likely to be networked. Moreover, paucity of data on the effects of cancer cachexia on heart muscle highlights the need to investigate this tissue in parallel to skeletal muscle for the purposes of identifying tissue-specific or global muscle responses to cancer cachexia.

We have recently undertaken a genomics approach to studying the multiple changes occurring in muscle during cancer cachexia [13]. While we were able to identify a broad variety of changes at the genomic level, whether these translate into functional changes depends on the realisation of downstream effects. Protein expression levels provide further information on whether the effects observed at the gene level are in fact translated, together with experimental validation of the observed genomic changes. Differential proteomics represents an effective analytical tool to gather information on multiple proteins in a single experimental setup. So far few studies have utilised this approach to investigate aspects of cancer: one study focused on the identification of urine biomarkers associated with cancer cachexia in patients and the other study reported the proteomic profile in skeletal muscle of cachectic rats [14, 15]. These MALDI-TOF experiments were either indirect (urinary proteins) or else focussed on protein oxidation/carbonylation specifically and precluded analysis of differentially expressed proteins. Two very recent studies on proteomic changes in cachexic muscle used label-free approaches [16, 17]. To our knowledge the current work represents the first use of iTRAQ quantitative mass spectrometry to investigate the proteomic profiles of cachexia affected skeletal (fast [gastrocnemius] and slow [soleus] twitch) and heart muscles. Isobaric tags for relative and absolute quantification (iTRAQ) followed by liquid chromatography-tandem mass spectrometry (LC-MS/MS) were used [18]. The iTRAQ approach allows multiple protein changes to be revealed at once as well as providing a validation tool for observed changes at the morphological and gene levels. Use of isobaric tags as internal standards provides higher data accuracy and reliability, and has been successfully applied to studies of proteomic changes associated with liver regeneration and tumour phenotypes of different cancers [19–22].

## RESULTS

### Proteomics analysis of different muscle types

**Figure 1:**
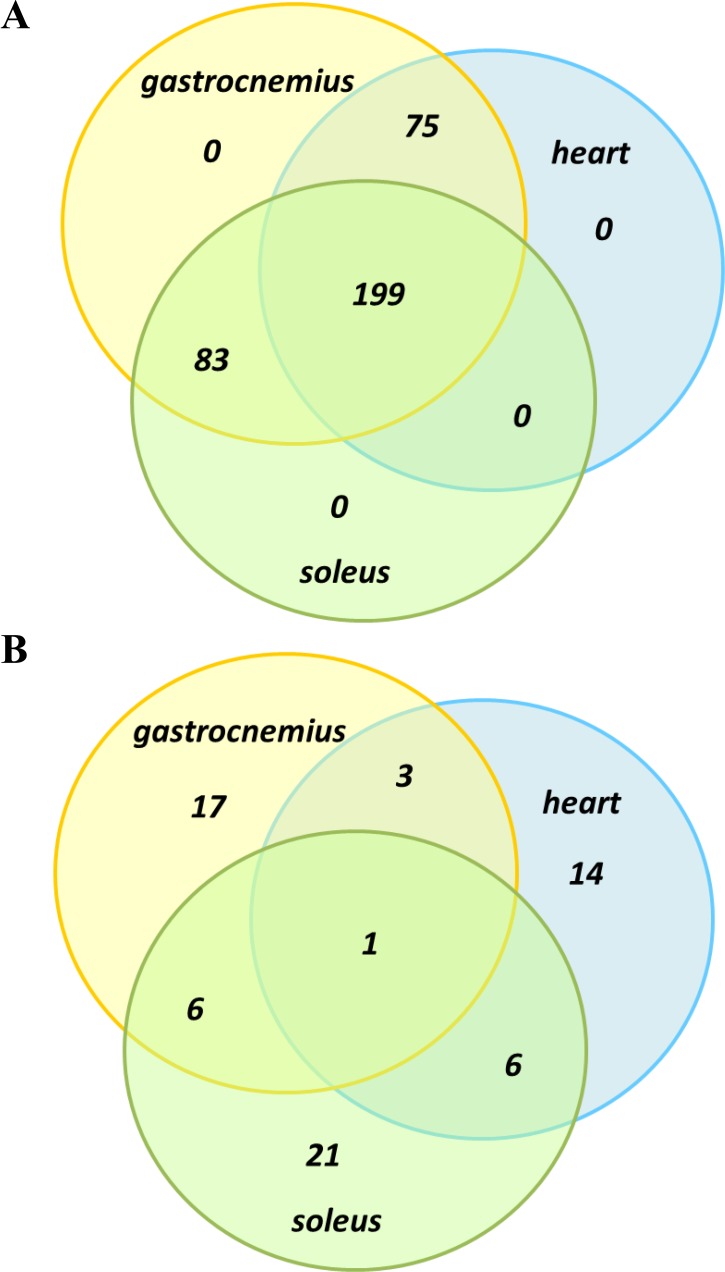
Venn diagram comparison of protein overlap between muscle groups Full protein lists, as shown in Supplementary Tables 1, 2 and 3, were compared (**A**) as well as significantly differentially expressed proteins lists, as shown in Tables 2, 3 and 4 (**B**).

Proteomics analysis identified and quantified more than 300 proteins, with > 95% confidence in correct sequence identification (unused score of > 1.3 and FDR of 1%), in each of two iTRAQ experiments. Spectral, peptide and protein summary data from the two experiments are shown in Table 1, and the full lists of proteins relating to each of the muscle types, gastrocnemius, soleus and heart are shown in the Supplementary Materials (Supplementary Tables 1, 2 and 3). Approximately 5–10% of proteins from the full lists were significantly differentially expressed, at a *p*-value of 0.05 (27 proteins from gastrocnemius, 34 proteins from soleus and 24 proteins from heart), and are shown in Tables 2, 3 and 4. When the full protein lists across muscle types were compared, 199 proteins were common across all three groups, 75 were common to gastrocnemius and heart, 83 were common to soleus and gastrocnemius, and no proteins were unique to just a single muscle type (Figure 1A). However, when a similar comparison was made based on the much shorter significantly differentially expressed protein lists (*i.e*., those shown in Tables 2, 3 and 4), then there was only a single protein that was common across all three muscle types (myosin-4 or Myh4), with 3–6 proteins common across pairs of muscle types, and the majority of differentially expressed proteins unique to each muscle type (Figure 1B).

**Table 1:** Summary data from Protein Pilot v5.0 for the two ITRAQ proteomics experiments

	iTRAQ Experiment 1	iTRAQ Experiment 2
**Spectra**	50,805	30,437
**Distinct peptides**	7,388	5,917
**Proteins (before grouping)**	2,659	1,434
**Proteins (after grouping)**	369	329
**Protein ID yield with Local FDR (1%)**	357	331
**Peptide ID yield with Local FDR (1%)**	4,333	3,196
**Spectral ID yield with Local FDR (1%)**	36,171	19,312

Data was processed with Protein Pilot™ 5.0 software using the Paragon™ Algorithm for the identification of spectra, peptides and proteins. The data represents a protein detection threshold cutoff > 1.3 (unused score), representing a confidence of correct sequence identification of > 95%.

**Table 2:** Proteins dysregulated in mouse gastrocnemius muscle during cancer cachexia, quantified by iTRAQ based discovery proteomics

Unused	%Cov(95)	gi Accession	Protein Name (protein term for STRING bioinformatics analysis)	Biol Rep	Peptides (95%)	cachexia/control iTRAQ tag area ratio	*P* Val
6.27	9.565	160358829	***hemopexin precursor (Hpx)***	BR3	4	1.935	0.00847
6.27	9.565			BR2	4	1.603	0.00119
25.65	36.520			BR5	16	1.565	0.00466
65.16	77.450	439253893	***actin, alpha skeletal muscle (Acta1)***	BR2	254	1.724	0.00718
65.16	77.450			BR3	254	1.515	0.04439
16.02	23.400	568909414	***myosin-binding protein H isoform X1 (MybpH)***	BR1	8	1.539	0.00461
16.02	23.400			BR3	8	1.233	0.03045
61.71	8.521	568913099	***nebulin isoform X9 (Nebl)***	BR3	38	1.500	0.00023
61.71	8.521			BR2	38	1.367	2.39E-07
61.71	8.521			BR1	38	1.257	3.21E-05
11.62	20.640	755519288	***troponin T, slow skeletal muscle isoform X2 (Tnnt1)***	BR3	8	1.282	0.02597
11.62	20.640			BR2	8	1.245	0.03467
27.66	72.180	11875203	***tropomyosin beta chain isoform Tpm2.2st [Tpm2]***	BR5	49	1.268	7.61E-05
123.22	92.610			BR3	116	1.136	0.00018
13.92	52.380	68226433	***histone H2B type 2-B (Hist2h2bb)***	BR3	9	1.250	0.00047
13.92	52.380			BR1	9	1.207	0.00161
163.01	77.090	205830428	***myosin heavy chain Iia (Myh2)***	BR3	604	1.208	0.04663
163.01	77.090			BR1	604	1.172	0.02887
163.01	77.090			BR2	604	1.134	0.00084
349.71	67.970	67189167	***myosin-4 [Myh4]***	BR5	254	1.189	2.01E-06
517.79	79.940			BR1	917	1.253	7.19E-05
517.79	79.940			BR2	917	1.073	0.01459
26.32	61.210	300069034	***PDZ and LIM domain protein 5 isoform ENH4 (Pdlim5)***	BR3	17	1.199	0.00136
26.32	61.210			BR2	17	1.146	0.02435
32.06	0.891	755499204	***titin isoform X3 (Ttn)***	BR1	23	1.177	0.03356
32.06	0.891			BR2	23	1.155	0.00572
204.45	67.600	18859641	***myosin-7 (Myh7)***	BR2	348	1.176	7.31E-09
204.45	67.600			BR1	348	1.111	0.00018
136.63	65.380	568947734	***myosin-binding protein C, fast-type isoform X1 [Mybpc2]***	BR1	79	1.159	5.04E-06
85.11	39.260			BR5	41	1.166	0.00515
75.05	61.190	157951643	***alpha-actinin-2 [Actn2]***	BR2	66	1.074	0.00484
75.05	61.190			BR1	66	1.091	0.02410
97.56	80.260	163310765	***serum albumin precursor [Alb]***	BR4	79	0.76	1.04E-09
95.64	79.610			BR1	65	0.94	0.05683
34.76	41.040	254553458	***glucose-6-phosphate isomerase (Gpi1)***	BR2	31	0.909	0.00992
34.76	41.040			BR1	31	0.855	0.02262
36.56	43.710	33563250	***desmin (Des)***	BR1	21	0.902	0.00300
36.56	43.710			BR2	21	0.894	0.00139
18.09	66.920	6753810	***fatty acid-binding protein, heart (Fabp3)***	BR4	26	0.891	0.04207
15.38	63.910			BR2	16	0.874	0.01664
65.9	81.680	6679937	***glyceraldehyde-3-phosphate dehydrogenase isoform 2 (Gapdh)***	BR2	96	0.888	0.03505
58.71	84.080			BR4	78	0.804	0.03664
52.99	48.750	227330633	***phosphoglucomutase-2 (Pgm2)***	BR2	32	0.888	0.00047
52.99	48.750			BR1	32	0.881	0.00351
63.56	45.130	18079339	***aconitate hydratase, mitochondrial precursor (Aco2)***	BR1	35	0.882	0.00050
63.56	45.130			BR2	35	0.882	0.00038
19.28	37.040	568912066	***ATP synthase subunit gamma, mitochondrial isoform X1 (Atp5c1)***	BR2	14	0.878	0.00898
19.41	33.670			BR5	11	0.819	0.00172
37.66	67.750	31982186	***malate dehydrogenase, mitochondrial precursor (Mdh2)***	BR1	26	0.875	0.00132
37.66	67.750			BR2	26	0.788	2.89E-06
138.32	68.170	6755256	***glycogen phosphorylase, muscle form (Pygm)***	BR1	103	0.863	9.37E-05
138.32	68.170			BR2	103	0.840	1.72E-08
34.34	54.360	148747424	***ADP/ATP translocase 1 (Slc25a4)***	BR1	23	0.826	0.00918
34.34	54.360			BR2	23	0.785	0.00854
12.45	32.390	568889869	***ATP synthase subunit O, mitochondrial [Atp5o]***	BR2	7	0.734	0.00203
10.26	27.700			BR4	6	0.802	0.00818
9.98	55.920	6679078	***nucleoside diphosphate kinase B [Nme2]***	BR1	8	0.752	0.00190
9.98	55.920			BR2	8	0.800	0.01394

**Figure 2:**
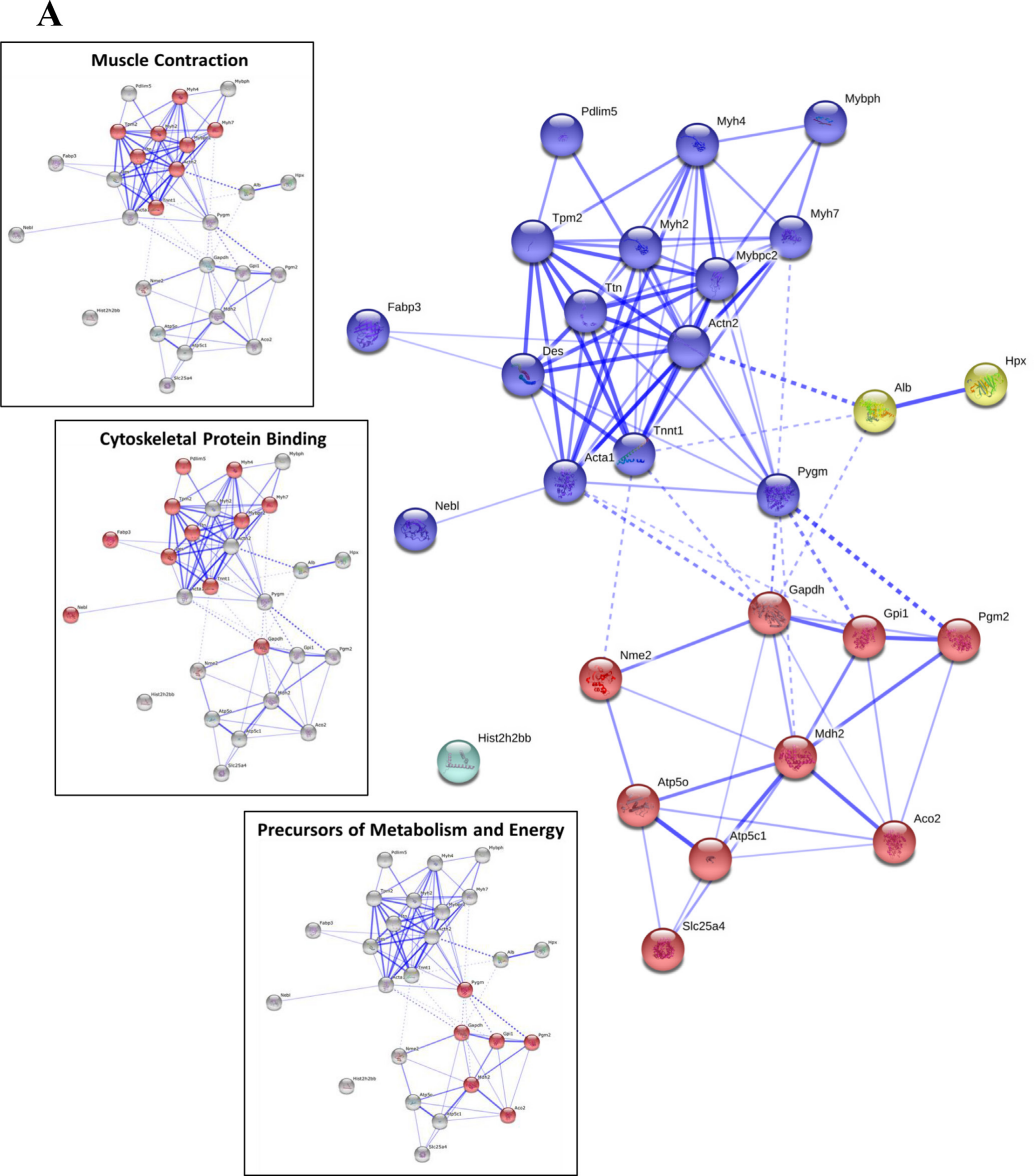

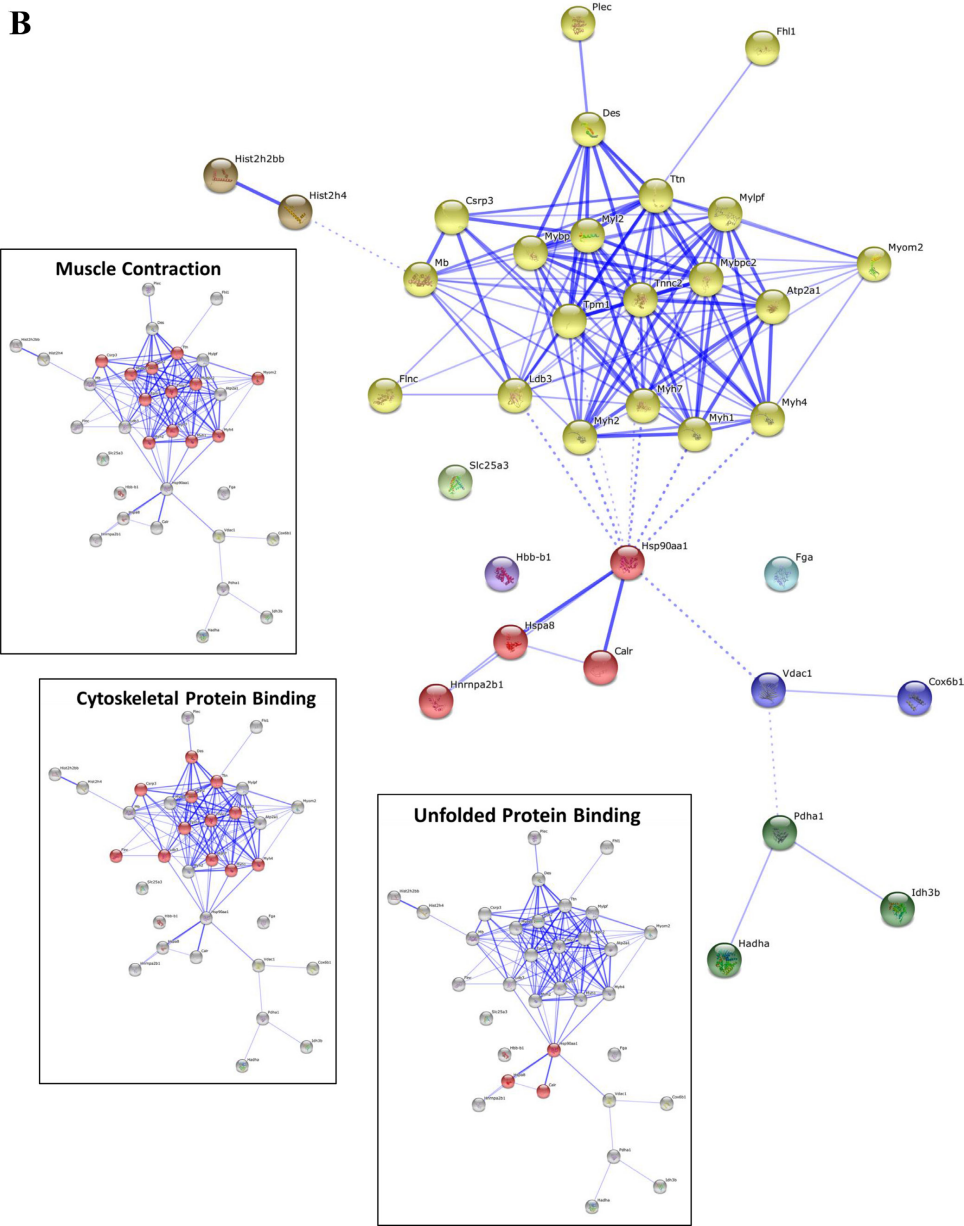

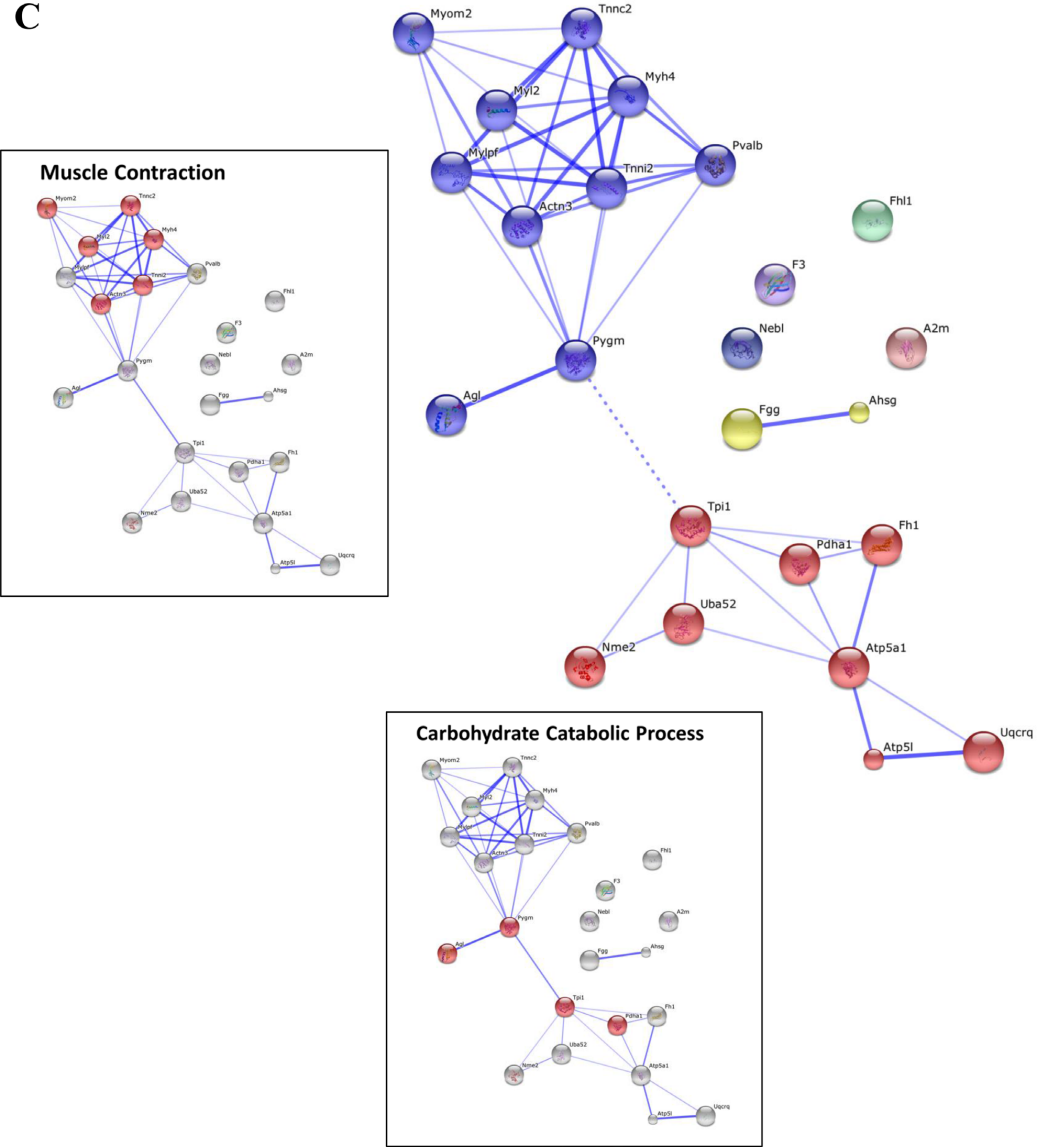
STRING v10 bioinformatics analysis of proteins dysregulated during cancer cachexia in gastrocnemius (27 proteins) (**A**), soleus (34 proteins) (**B**) and heart (24 proteins) (**C**) muscle. MCL 2 point clustering was used for the STRING analysis, and the differentially expressed proteins used for clustering are shown in Tables 2, 3 and 4. The insets show some of the significantly enriched biological processes and molecular functions highlighted in red against grey clusters (the full list of significant enrichment is shown in Table 5).

Two technical replicates (*n* = 2) and five biological replicates (*n* = 5) therefore *n* = 10 samples were used and a total of 377 proteins identified and quantified by 2D LCMSMS (QStar Elite) and ProteinPilot v5.0 analysis. The proteins which were differentially expressed in a consistent direction in the majority of biological replicates, and that were also statistically significant in two or more biological replicates, are listed in this table (27 proteins in all). The full table of identified proteins is provided in Supplementary Table 1. This short-list of differentially expressed proteins was processed using the STRING v10 WEB based bioinformatics tool, and the identified clusters and protein enrichment shown in Figure 2A and Table 5 respectively.

**Table 3:** Proteins dysregulated in mouse soleus muscle during cancer cachexia, quantified by iTRAQ based discovery proteomics

Unused	%Cov(95)	gi Accession	Name (protein term for STRING bioinformatics analysis)	Peptides (95%)	cachexia/control iTRAQ tag area ratio	*P* Val
2.33	1.648	167555029	***fibrinogen alpha chain isoform 1 precursor (Fga)***	1	7.622	0.03395
163.01	77.090	205830428	***myosin heavy chain Iia (Myh2)***	604	2.317	7.33E-09
36.51	66.550	116517336	***four and a half LIM domains protein 1 isoform 2 (Fhl1)***	26	2.190	2.49E-06
204.45	67.600	18859641	***myosin-7 (Myh7)***	348	2.179	7.61E-14
10.47	16.640	6754254	***heat shock protein HSP 90-alpha (Hsp90aa1)***	15	2.108	0.00198
5.09	21.650	7304987	***cysteine and glycine-rich protein 3 (Csrp3)***	3	1.949	0.04617
35.4	30.930	84875544	***LIM domain-binding protein 3 isoform c (Ldb3)***	29	1.947	0.00235
52.65	26.890	568966329	***myosin-binding protein C, slow-type isoform X14 (Mybpc2)***	32	1.878	6.31E-07
6.76	10.760	755517050	***heterogeneous nuclear ribonucleoproteins A2/B1 isoform X1 (Hnrnpa2b1)***	5	1.766	0.00248
40.83	9.432	568939784	***filamin-C isoform X1 (Flnc)***	23	1.755	0.00031
36.56	43.710	33563250	***desmin (Des)***	21	1.648	4.46E-05
7.46	22.600	6680836	***calreticulin precursor (Calr)***	5	1.493	0.01178
13.92	52.380	68226433	***histone H2B type 2-B (Hist2h2bb)***	9	1.487	0.00297
55.41	77.090	82524274	***myosin-1 (Myh1)***	648	1.483	0.00235
32.06	0.891	755499204	***titin isoform X3 (Ttn)***	23	1.333	0.00746
41.06	37.460	31981690	***heat shock cognate 71 kDa protein (Hspa8)***	24	1.330	0.02401
13.5	2.518	254675244	***plectin isoform 1 (Plec)***	9	1.324	0.02871
73.3	90.850	256000780	***tropomyosin alpha-1 chain isoform Tpm1.1st (Tpm1)***	134	1.316	0.00037
517.79	79.940	67189167	***myosin-4 (Myh4)***	917	1.278	1.73E-05
27.09	65.000	6678371	***troponin C, skeletal muscle (Tnnc2)***	39	1.254	0.02125
127.94	42.870	568953176	***myomesin-2 isoform X2 (Myom2)***	78	1.208	0.00377
136.63	65.380	568947734	***myosin-binding protein C, fast-type isoform X1 (Mybpc2)***	79	1.207	0.00239
131.92	55.140	568949470	***sarcoplasmic/endoplasmic reticulum calcium ATPase 1 isoform X1 (Atp2a1)***	106	1.160	0.03768
13.31	20.390	568966988	***phosphate carrier protein, mitochondrial isoform X1 (Slc25a3)***	9	0.751	0.01161
9.46	13.540	18700024	***isocitrate dehydrogenase 3, beta subunit (Idh3b)***	5	0.741	0.02699
12.9	20.000	6679261	***pyruvate dehydrogenase E1 component subunit alpha, somatic form, mitochondrial precursor (Pdha1)***	9	0.740	0.02822
31.64	24.640	33859811	***trifunctional enzyme subunit alpha, mitochondrial precursor (Hadha)***	15	0.728	0.00343
53.27	95.270	7949078	***myosin regulatory light chain 2, skeletal muscle isoform (Mylpf)***	114	0.717	0.00636
20.82	78.910	498752597	***hemoglobin subunit beta-1 (Hbb-b1)***	18	0.646	0.00448
18.25	44.170	6755963	***voltage-dependent anion-selective channel protein 1 (Vdac1)***	9	0.625	0.03542
3.5	20.930	13385090	***cytochrome c oxidase subunit 6B1 (Cox6b1)***	3	0.589	0.00206
40.28	88.960	255708425	***myoglobin (Mb)***	45	0.387	0.00094
13.55	53.400	568893484	***histone H4 (Hist2h4)***	12	0.305	0.00360
26.34	77.720	568936906	***myosin regulatory light chain 2, ventricular/cardiac muscle isoform isoform X1 (Myl2)***	21	0.282	1.15E-06

Two technical replicates (*n* = 2) and a single biological replicate (*n* = 1), therefore *n* = 2 samples were used. A total of 285 proteins were identified and quantified by LCMSMS (QStar Elite) and ProteinPilot v5.0 analysis. Significantly dysregulated proteins are listed in this table (34 proteins). The full table of identified/quantified proteins is provided in Supplementary Table 2. This list of proteins was also processed using the STRING v10 WEB based bioinformatics tool, and the identified clusters and protein enrichment shown in Figure 2B and Table 5 respectively.

**Table 4:** Proteins dysregulated in mouse heart muscle during cancer cachexia, quantified by iTRAQ based discovery proteomics

Unused	%Cov(95)	gi Accession	Protein Name (protein term for STRING bioinformatics analysis)	Biol Rep	Peptides (95%)	cachexia/control iTRAQ tag area ratio	*P* Val
2.71	4.358	19527078	***fibrinogen gamma chain precursor (Fgg)***	BR2	2	5.434	0.02153
3.87	10.43	7304875	***alpha-2-HS-glycoprotein isoform 1 precursor (Ahsg)***	BR2	3	1.698	0.02759
42.25	37.59	20330802	***serotransferrin precursor (Tf)***	BR2	24	1.558	1.66E-06
55.67	96.36	568991776	***parvalbumin alpha isoform X1 (Pvalb)***	BR1	60	1.556	1.46E-05
5.38	36.59	568973615	***cytochrome b-c1 complex subunit 8 isoform X1 (Uqcrq)***	BR1	5	1.539	0.02216
18.58	2.393	568913135	***nebulin isoform X26 (Nebl)***	BR1	11	1.445	2.77E-05
7.68	22.5	568384815	***four and a half LIM domains protein 1 isoform 3 (Fhl1)***	BR1	5	1.393	0.04835
22.73	8.355	568924485	***glycogen debranching enzyme isoform X1 (Agl)***	BR1	12	1.334	0.03097
25.23	62.09	6678391	***troponin I, fast skeletal muscle (Tnni2)***	BR1	21	1.310	0.02100
7.9	4.348	110347469	***alpha-2-macroglobulin precursor (A2m)***	BR2	6	1.306	0.03769
349.71	67.97	67189167	***myosin-4 (Myh4)***	BR1	254	1.290	1.01E-08
22.03	65	6678371	***troponin C, skeletal muscle (Tnnc2)***	BR1	31	1.263	0.00660
22.03	65	6678371		BR2	31	1.175	0.03371
44.94	95.27	7949078	***myosin regulatory light chain 2, skeletal muscle isoform (Mylpf)***	BR1	63	1.223	0.00132
90.83	50.89	7304855	***alpha-actinin-3 (Actn3)***	BR1	46	1.216	0.00119
34.69	64.88	226958349	***triosephosphate isomerase (Tpi1)***	BR2	26	1.180	0.02520
119.92	71.97	6755256	***glycogen phosphorylase, muscle form (Pygm)***	BR1	106	1.165	0.00628
77.95	33.24	568953176	***myomesin-2 isoform X2 (Myom2)***	BR1	40	1.147	0.02854
77.95	33.24	568953176		BR2	40	1.104	0.04321
71.57	55.88	6680748	***ATP synthase subunit alpha, mitochondrial precursor (Atp5a1)***	BR1	45	0.813	0.01146
71.57	55.88	6680748		BR2	45	0.896	0.02872
17.54	28.21	6679261	***pyruvate dehydrogenase E1 component subunit alpha, somatic form, mitochondrial precursor (Pdha1)***	BR2	10	0.861	0.00378
31.14	33.73	226823367	***fumarate hydratase, mitochondrial precursor (Fh)***	BR2	19	0.815	0.00233
10.46	46.09	9845265	***ubiquitin-60S ribosomal protein L40 (Uba52)***	BR1	6	0.722	0.00579
10.46	46.09	9845265		BR2	6	0.813	0.00761
13.34	55.92	6679078	***nucleoside diphosphate kinase B (Nme2)***	BR1	9	0.752	0.00838
13.34	55.92	6679078		BR2	9	0.719	0.02891
4.08	27.18	31980744	***ATP synthase subunit g, mitochondrial (Atp5l)***	BR2	2	0.676	0.04705
27.32	73.27	568936906	***myosin regulatory light chain 2, ventricular/cardiac muscle isoform isoform X1 (Myl2)***	BR1	30	0.671	0.00436

Two technical replicates (*n* = 2) and two biological replicates (*n* = 2), therefore *n* = 4 samples were used and a total of 278 proteins identified and quantified by 2D LCMSMS (QStar Elite) and ProteinPilot v5.0 analysis. The proteins which were significantly differentially expressed and in a consistent direction, in both biological replicates, are listed in this table (24 proteins). The full table of identified proteins is provided in Supplementary Table 3. This list of proteins was also processed using the STRING v10 WEB based bioinformatics tool, and the identified clusters and protein enrichment shown in Figure 2C and Table 5 respectively.

### Bioinformatics analysis

**Figure 3:**
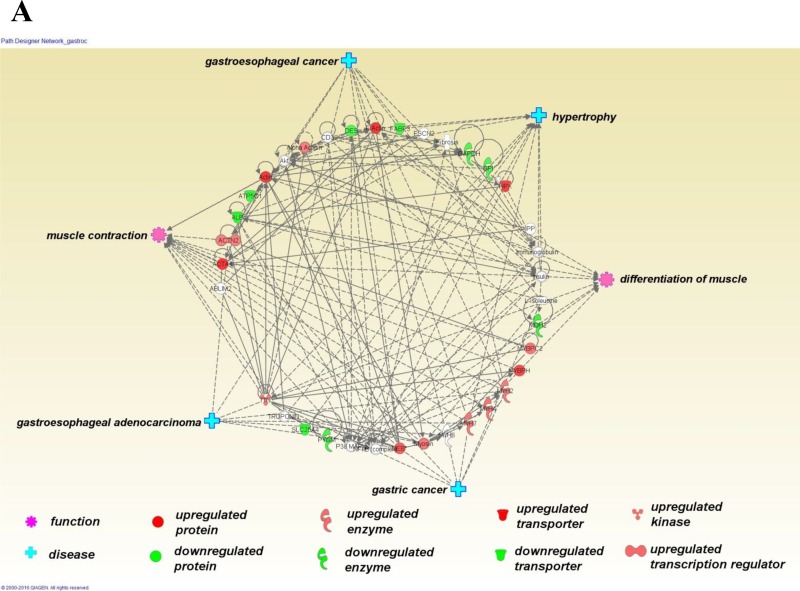

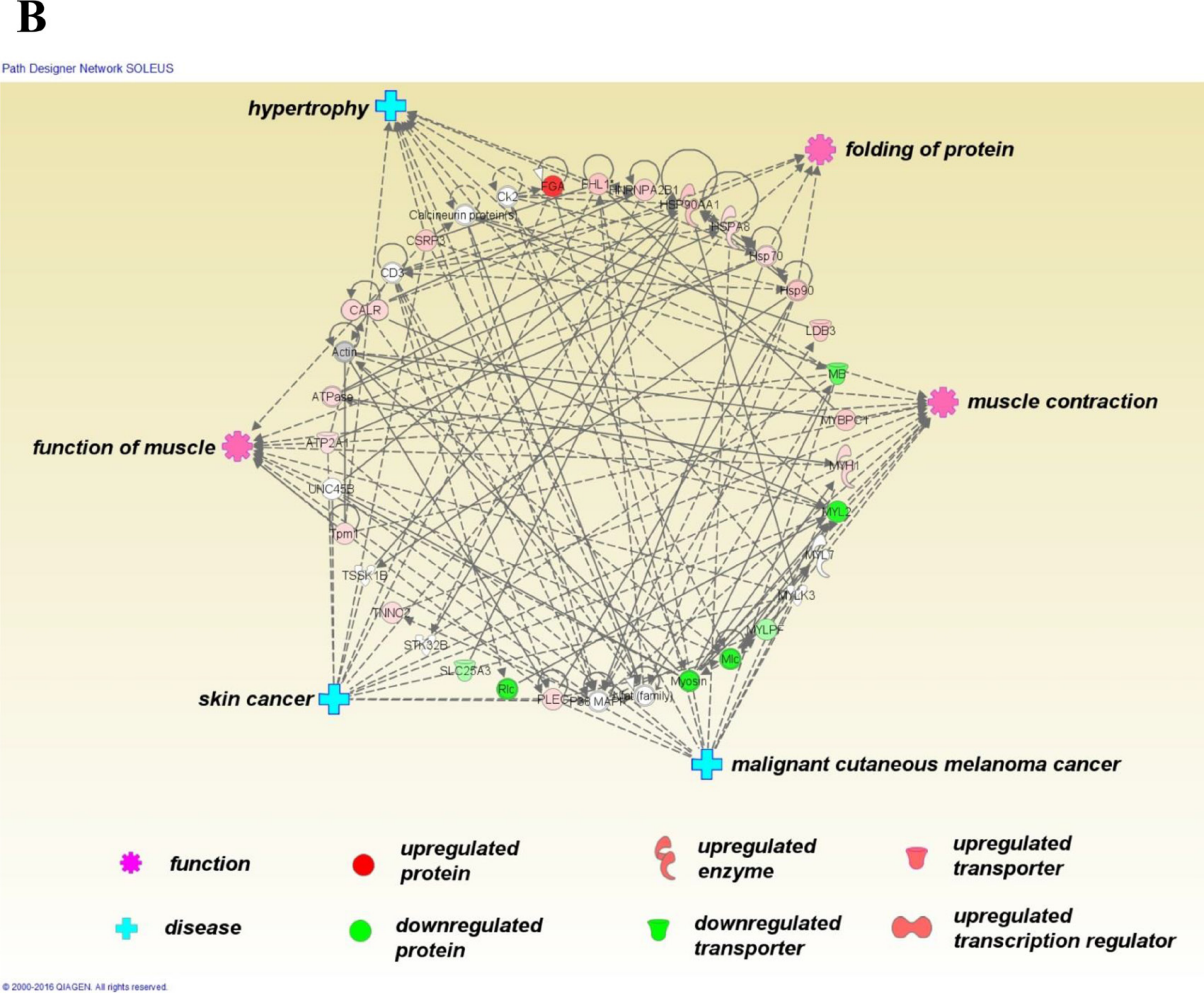

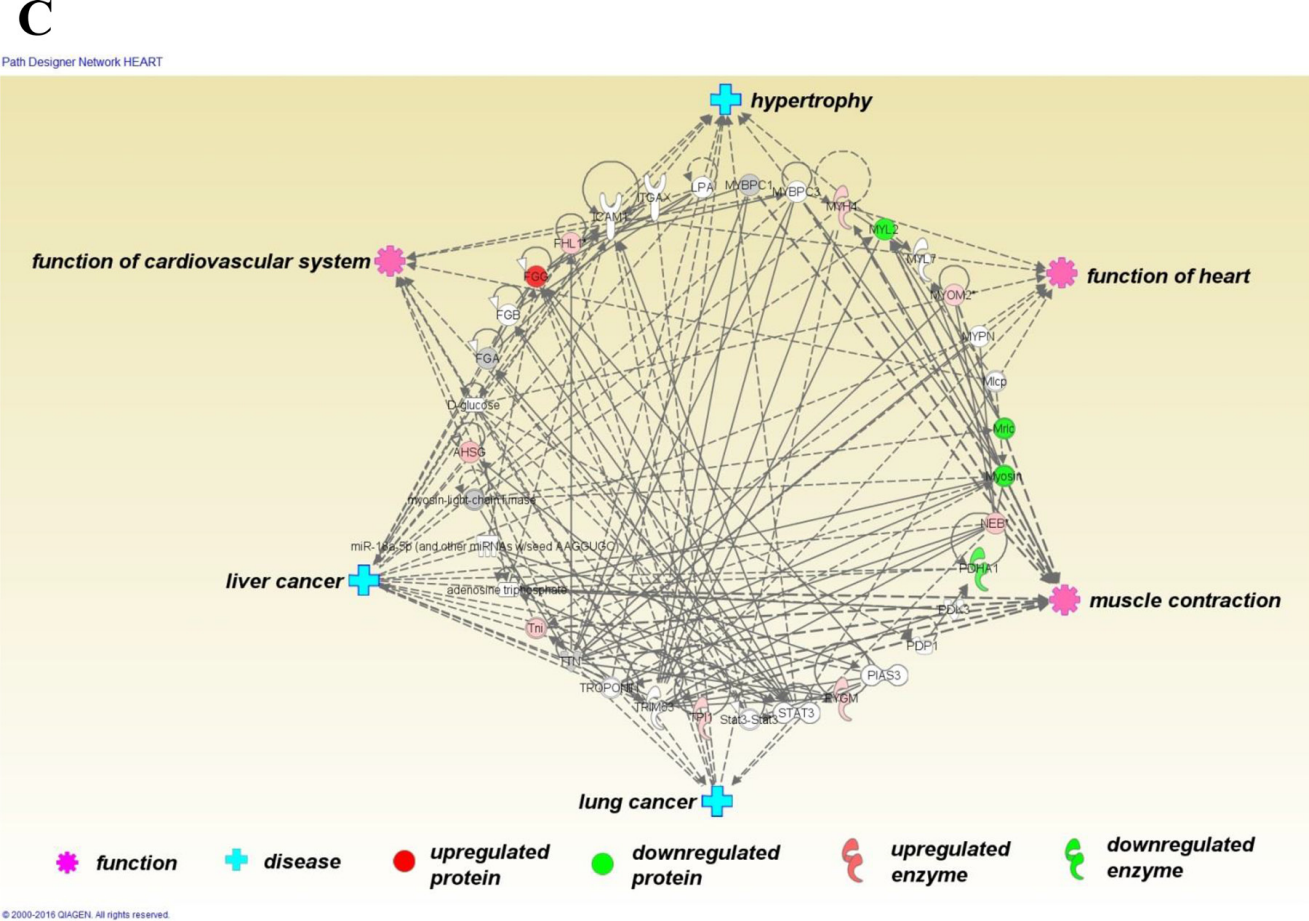
Ingenuity Pathway Analysis (Qiagen 2015) was used to explore relationships of the differentially expressed proteins to each other and to diseases and functions Differentially expressed proteins (derived from the lists shown in Tables 2, 3 and 4) are shown in a circular layout (upregulated proteins in red, downregulated proteins in green and non-coloured molecules predicted by the software to have interactions). Six statistically significantly related functions (pink cog) and diseases (blue cross) are displayed at points around the central circle. The top ID associated network identified by IPA for each of the muscle types is shown in this figure; gastrocnemius (**A**), soleus (**B**) and heart (**C**). For gastrocnemius, the most significant ID associated network functions (IPA top network score = 53) are skeletal and muscular system development and function, cardiovascular disease, organismal injury and abnormalities (IPA statistically significant diseases and functions sub-categories frequently show cancers of various kinds). For soleus, the most significant ID associated network functions (IPA top network score = 44) are skeletal and muscular system development and function, cardiovascular system development and function, embryonic development. For heart, the most significant ID associated network functions (IPA top network score = 21) were skeletal and muscular system development and function, cardiovascular system development and function, cellular assembly and organization.

**Figure 4:**
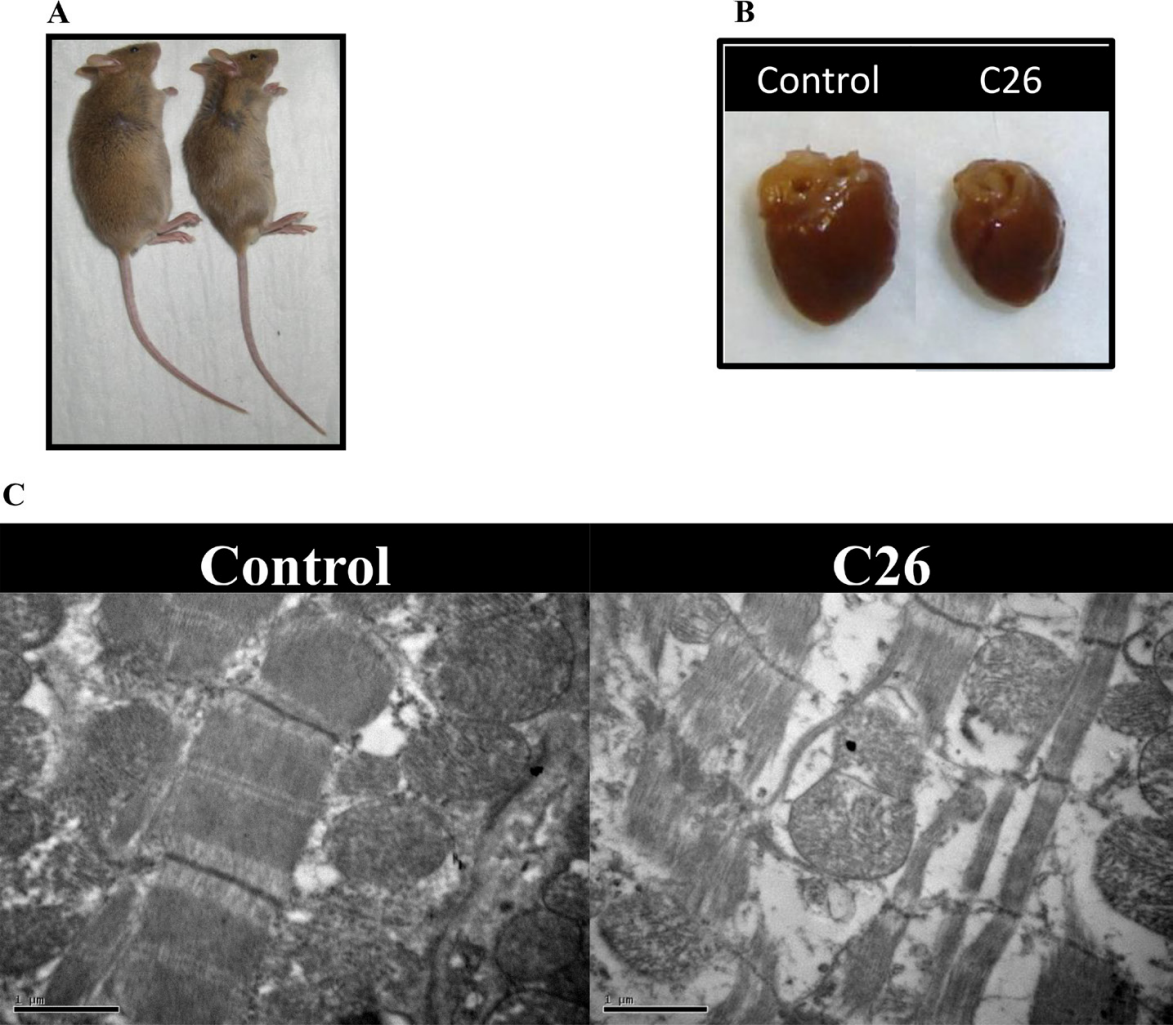

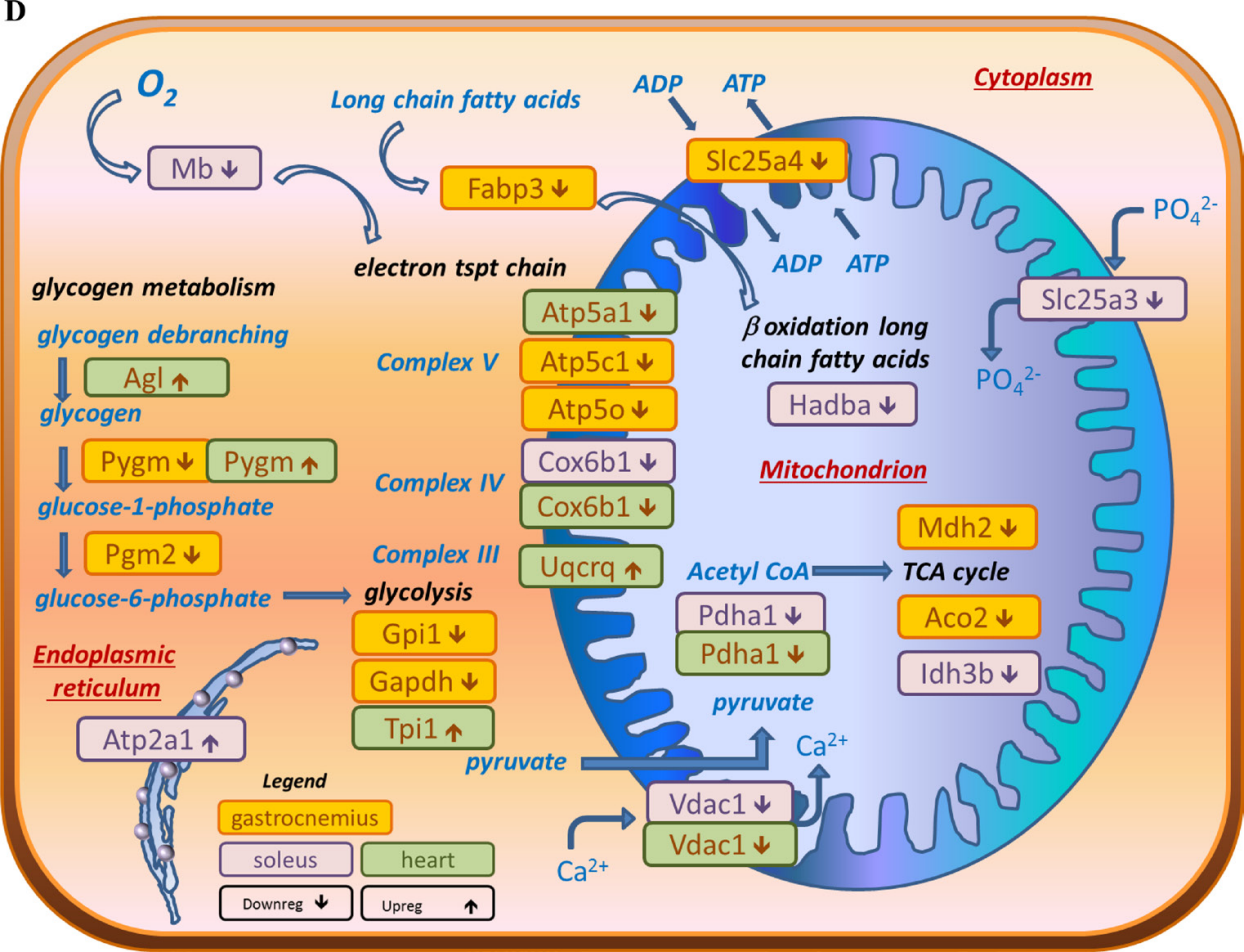
(**A**) Tumour-mediated effect on whole body size. (**B**) Tumour-mediated effect on heart. (**C**) Electron micrographs showing structural and mitochondrial alterations in cardiac muscle of NTB control and C26-bearing mice. Increased intercellular spaces were seen and appeared to be associated with a loss of myofibrils and reduced density of mitochondrial matrix (Magnification = 25,000×). (**D**) Schematic of the role of muscle proteins differentially expressed during cancer-induced cachexia in the cellular energy/metabolism pathways. Differentially expressed proteins are indicated as small rectangles, with colour representing the muscle type in which differential expression was observed (yellow = gastrocnemius, purple = soleus, green = heart) and the arrows indicating up (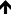) or down (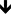) regulation. The displayed proteins were those identified in the proteomics experiments and listed in Tables 2, 3 and 4.

The significantly differentially expressed protein lists (*i.e*., Tables 2, 3 and 4) were used to explore enrichment of gene ontology (GO) biological processes and molecular functions (Table 5), and protein clusters (Figure 2). Three main processes or functions emerged across muscle types; muscle contraction, metabolites/energy and cytoskeletal protein binding (Table 5). A few processes/functions were uniquely identified in just a single muscle type, including; unfolded protein binding (soleus) and complement and coagulation cascades (heart). Protein group clustering is shown in Figure 4 for each muscle type, with the specific protein involved in enriched biological processes and molecular functions highlighted. In general, muscle contraction and cytoskeletal binding proteins were upregulated in the C26 cachectic mice, whereas the energy/metabolism protein group was generally downregulated in the C26 mice (Tables 2, 3, 4). Ingenuity Pathway Analysis (IPA) was used to explore relationships of the differentially expressed proteins to each other and to diseases and functions (Figure 3). The top ID associated network identified by IPA in each of the muscle types was “skeletal and muscular system development and function”. Other networks were also identified for each of the specific muscle types, including; organismal injury and abnormalities in gastrocnemius (IPA statistically significant diseases and functions sub-categories frequently show cancers of various kinds). For soleus, additional significant ID associated networks included cardiovascular system development and function and embryonic development. For heart, additional significant ID associated network functions included cardiovascular system development and function and cellular assembly and organization.

**Table 5:** Enrichment of biological processes, molecular functions and KEGG pathways of protein lists that are differentially expressed in mouse gastrocnemius, soleus and heart muscle during cancer cachexia

		GO id	Term	Number of Genes	*p*-value	*p*-value FDR	*p*-value Bonferroni
**Gastrocnemius**	**Biological Process Enrichment**	GO:0006936	muscle contraction	8	2.59E-12	3.48E-08	3.48E-08
		GO:0003012	muscle system process	8	2.20E-11	1.48E-07	2.96E-07
		GO:0006091	generation of precursor metabolites and energy	6	6.77E-08	3.04E-04	9.11E-04
		GO:0044724	single-organism carbohydrate catabolic process	4	8.49E-07	2.85E-03	1.14E-02
		GO:0016052	carbohydrate catabolic process	4	1.31E-06	3.52E-03	1.76E-02
	**Molecular Function Enrichment**	GO:0008092	Cytoskeletal protein binding	11	1.46E-10	6.89E-07	6.89E-07
		GO:0003779	actin binding	7	8.28E-08	1.95E-04	3.89E-04
		GO:0005200	structural constituent of cytoskeleton	4	4.85E-07	7.60E-04	2.28E-03
		GO:0046933	proton-transporting ATP synthase activity, rotational mechanism	2	3.81E-05	4.48E-02	1.79E-01
	**KEGG Pathways Enrichment**	4530	Tight junction	4	1.84E-05	5.24E-03	5.24E-03
		5410	Hypertrophic cardiomyopathy (HCM)	3	1.30E-04	1.46E-02	3.69E-02
		5414	Dilated cardiomyopathy	3	1.55E-04	1.46E-02	4.39E-02
**Soleus**	**Biological Process Enrichment**	GO:0003012	muscle system process	13	1.91E-19	2.57E-15	2.57E-15
		GO:0006936	muscle contraction	12	5.93E-19	3.98E-15	7.97E-15
		GO:0006941	striated muscle contraction	6	8.75E-10	3.92E-06	1.18E-05
		GO:0030029	actin filament-based process	9	2.10E-09	7.05E-06	2.82E-05
		GO:0007517	muscle organ development	7	6.10E-08	1.64E-04	8.20E-04
		GO:0061061	muscle structure development	8	1.22E-07	2.73E-04	1.64E-03
		GO:0060048	cardiac muscle contraction	4	5.49E-07	9.98E-04	7.38E-03
		GO:0030239	myofibril assembly	4	6.02E-07	9.98E-04	8.09E-03
		GO:0055002	striated muscle cell development	5	6.68E-07	9.98E-04	8.98E-03
		GO:0055001	muscle cell development	5	1.25E-06	1.52E-03	1.68E-02
		GO:0055003	cardiac myofibril assembly	3	1.39E-06	1.52E-03	1.86E-02
		GO:0003008	system process	14	1.43E-06	1.52E-03	1.92E-02
		GO:0060047	heart contraction	4	1.47E-06	1.52E-03	1.97E-02
		GO:0055008	cardiac muscle tissue morphogenesis	4	1.94E-06	1.86E-03	2.61E-02
		GO:0003015	heart process	4	2.07E-06	1.86E-03	2.79E-02
		GO:0070252	actin-mediated cell contraction	3	2.94E-06	2.47E-03	3.95E-02
		GO:0014706	striated muscle tissue development	6	3.18E-06	2.52E-03	4.28E-02
		GO:0060415	muscle tissue morphogenesis	4	3.61E-06	2.66E-03	4.85E-02
	**Molecular Function Enrichment**	GO:0005200	structural constituent of cytoskeleton	7	1.49E-12	7.00E-09	7.00E-09
		GO:0003779	actin binding	10	3.19E-11	7.49E-08	1.50E-07
		GO:0008092	cytoskeletal protein binding	12	1.55E-10	2.42E-07	7.27E-07
		GO:0008307	structural constituent of muscle	4	9.90E-09	1.13E-05	4.66E-05
		GO:0005198	structural molecule activity	9	1.21E-08	1.13E-05	5.67E-05
		GO:0032403	protein complex binding	9	7.30E-06	5.73E-03	3.44E-02
		GO:0042805	actinin binding	3	1.10E-05	7.37E-03	5.16E-02
		GO:0031433	telethonin binding	2	1.31E-05	7.69E-03	6.15E-02
		GO:0051371	muscle alpha-actinin binding	2	6.08E-05	3.18E-02	2.86E-01
		GO:0005516	calmodulin binding	4	1.01E-04	4.75E-02	4.75E-01
		GO:0051082	unfolded protein binding	3	1.15E-04	4.92E-02	5.42E-01
	**KEGG Pathways Enrichment**	4530	Tight junction	6	4.48E-08	1.27E-05	1.27E-05
		5410	Hypertrophic cardiomyopathy (HCM)	5	1.49E-07	1.89E-05	4.23E-05
		5414	Dilated cardiomyopathy	5	2.00E-07	1.89E-05	5.68E-05
		4260	Cardiac muscle contraction	4	4.75E-06	3.38E-04	1.35E-03
		4612	Antigen processing and presentation	3	1.71E-04	9.73E-03	4.87E-02
**Heart**	**Biological Process Enrichment**	GO:0006936	muscle contraction	6	4.92E-09	6.61E-05	6.61E-05
		GO:0003012	muscle system process	6	2.41E-08	1.62E-04	3.24E-04
		GO:0044724	single-organism carbohydrate catabolic process	4	5.17E-07	2.32E-03	6.96E-03
		GO:0016052	carbohydrate catabolic process	4	7.98E-07	2.68E-03	1.07E-02
	**Molecular Function Enrichment**	GO:0003779	actin binding	5	2.03E-05	9.53E-02	9.53E-02
	**KEGG Pathways Enrichment**	4530	Tight junction	4	1.13E-05	3.21E-03	3.21E-03
		4610	Complement and coagulation cascades	3	6.97E-05	9.89E-03	1.98E-02

Proteins were identified and quantified by iTRAQ based discovery proteomics (see Tables 2, 3 and 4) and processed using the STRING v10 WEB based bioinformatics tool to identify protein enrichment categories. Cellular compartment enrichment is shown in the supplementary materials (Supplementary Table 4).

### Electron microscopy of cardiac muscle

#### Sarcomere structure

Disrupted ultrastructural features were evident in cardiac muscle from C26-bearing mice. Hearts from C26-bearing mice were significantly smaller and the absolute weight was less than hearts from NTB controls (Table 6, Figures 4A and 4B). Analysis of muscle from control mice using electron microscopy showed the parallel register of myofibrils consisting of sarcomeres in series (Figure 4C). The sarcomere is the functional and structural unit for contraction and is demarcated by two Z-discs. Sections from cachectic muscle harvested at the endpoint (20% weight loss, day 14) exhibited altered ultrastructure to varying degrees in different myocytes. Vesicle-like structures were apparent at the Z-discs, and invaded into surrounding myofibrils [23]. Sarcomere disintegration was also evident, and extreme changes seen where the sarcomeres appeared ‘torn’ at the M-line (Figure 4C).

**Table 6:** Weight of carcass (whole body minus tumor) and tumor; absolute and normalized weights of heart at the endpoint

	Carcass Weight (g)	TumourWeight (g)	Heart Weight (mg)
**Control**	27 ± 0.3	---	130.6 ± 1.8
**C26**	20 ± 0.2 ^***^	0.7 ± 0.1	107.4 ± 2.6 ^**^

Significant weight loss was observed in C26-bearing mice on day 14 after tumor inoculation (endpoint). Absolute heart weight was reduced in C26-bearing mice. Statistical significance of weight was determined by two-tailed unpaired *t*-test, ^*^p < 0.05, ^**^p < 0.01, ^***^p < 0.001 compared to the controls (*n* = 4).

Mitochondrial structure: The normal appearance of mitochondria with densely packed cristae and homogenous matrix was diminished in muscles of C26-bearing mice (Figure 4C). Mitochondrial changes such as the presence of electron-lucent areas and swelling are evident. Some mitochondria are associated with vesicle-like structures which may represent autophagic bodies in the myocyte, with a reduced proportion of normal mitochondria observed. By contrast, the proportion of mitochondria with locally electron-lucent matrix increased in the C26 cachectic group and swelling and/or fragmentation features of cristae were more apparent in mitochondria of C26-bearing mice compared to the controls (Figure 4C).

These changes in mitochondria were observed to be similar to the features seen in the electron micrographs of cachectic skeletal muscle described in our previous work [24], and were consistent with the proteomic changes observed in both the myofibril/cytoskeletal and the energy/metabolism protein groups. When the energy/metabolism group of differentially expressed proteins were superimposed on the various cellular metabolic pathways (Figure 4D), it was evident that multiple metabolic pathways were affected by downregulated protein expression, including glycogen metabolism, glycolysis, electron transport chain, TCA cycle and fatty acid metabolism/β oxidation. Furthermore, several proteins involved in transport of ions and ADP/ATP across the mitochondrial lumen were also downregulated (Vdac1, Slc25a3 and Slc25a4). Proteins from the gastrocnemius have the largest number of differentially expressed proteins related to energy/metabolism, and are represented across all of the metabolic pathways (Figure 4D).

## DISCUSSION

This work represents the first study into muscle proteomic changes in the C26 mouse model of cancer cachexia. It follows our published genomics study on the same model [13] and has the further advantage that we were able to identify downstream effects in the form of protein expression changes relating to muscle function (*i.e*., functional genomics). Furthermore, effects across slow twitch, fast twitch and heart muscle types have been compared.

### Proteomic similarities and differences across muscle types

In all muscle samples, the majority of differentially expressed proteins fell into three main groups; muscle function, energy/metabolism and cytoskeleton. A small number of proteins had muscle specific enrichment, such as proton transporting ATP synthase activity in gastrocnemius, unfolded protein binding in soleus and complement and coagulation cascades in cardiac muscle (Table 5).

There was considerable overlap of the full list of proteins identified across the three muscle types (199 proteins identified across all three muscle types). However, there was very little overlap of specific proteins that were differentially expressed (see Venn diagrams in Figure 1A and 1B). Even though muscle function and energy/metabolism processes were represented across all three muscle types, only one differentially expressed protein occurred across all three muscle types (Myh4, upregulated in all 3 muscle types) and just a small amount of overlap was found between each of the muscle types (Figure 1B). Three proteins overlapped between gastrocnemius and heart and these were: Nebl (upregulated in both), Nme2 (downregulated in both) and Pygm (upregulated in heart and downregulated in gastrocnemius). Between soleus and heart, six proteins were differentially expressed in both: Fhl1, Myom2 and Tnnc2 being upregulated in both muscle types, Pdha1 and Myl2 being downregulated in both and Mylpf upregulated in heart and downregulated in soleus. In soleus and gastrocnemius six proteins overlap, with upregulation of Hist2h2bb, Mybpc2, Myh2, Myh7 and Ttn in both muscle types, with Des upregulated in soleus but downregulated in gastrocnemius. Nonetheless, most of the differentially expressed proteins across muscle types were involved in the same pathways/processes. This is well illustrated in Figure 4D which shows common metabolic pathways affected across muscle types, even though specific proteins within these pathways may differ.

### The sarcomere and disruption of muscle architecture in cancer cachexia

Loss of sarcomeres, disintegration of Z-discs and swollen mitochondria have been reported in skeletal muscle of sepsis- or denervation-induced myopathies [25, 26]. The molecular structure and components of the Z-disc have previously been described [27], and consist largely of titin, α-actinin and nebulin. Since the Z-disc is an anchor point for myofibrillar proteins such as myosin (via large protein titin) and actin, disrupted Z-discs could result in the release of these myofibrillar proteins. This was evident in a sepsis model with higher myofibrillar protein release rate [25]. In this study, all of the main Z-disc proteins were identified in the proteomics lists (Tables 2–4), and were consistently upregulated across all three muscle types, likely reflecting the disintegration of the Z-disc and release of its constituent proteins. There is a possibility that this upregulation of sarcomeric proteins in the soluble extracts is due to upregulated gene expression, however unlikely for the following reasons: (1) our data represent comparisons of C26 with control muscle samples that underwent identical extraction processes. By comparison with the soluble protein fraction, and even membrane proteins, the normal/intact sarcomere is a robust structure, containing some particularly high molecular weight proteins and protein complexes which are difficult to extract [28, 29]. So weakening of the sarcomere in diseases/disorders such as muscular dystrophy, cachexia and sarcopaenia are likely to facilitate extraction of sarcomere associated proteins. In fact muscle wasting in cachexia has previously been causally linked to proteasome-mediated protein degradation [30], (2) cancer cachexia is a catabolic rather than an anabolic process with weakened sarcomeres and muscle ultrastructural abnormalities, as supported by the electron microscopy data (Figure 4C), our previous work [24] and also data from other groups [30–33], (3) our previous genomics study has identified upregulation of protein catabolic processes, particularly those related to ubiquitin [13], and increased protein ubiquitination has also been reported by others [30] so degradation of muscle proteins is likely at play – which would also be consistent with facilitating release/upregulation of sarcomeric proteins during the protein extraction protocol, by comparison with normal controls, (4) gene expression of some muscle structural proteins, such as troponin are reduced in cachexia [33, 34], likely further weakening the sarcomere. We therefore argue that the balance of evidence supports the likelihood that upregulation of sarcomere associated peptides in the C26 samples indicates sarcomere breakdown rather than a homeostatic response to sarcomere weakening. This is the simplest explanation given that the key feature of cachexia is muscle breakdown. It aligns with our observation which is methodologically consistent with extraction being easier and more peptides now appearing in solution upon extraction. Moreover, the M-line has also been suggested to be an important site for the regular register of thick filaments and contributes to the stability of sarcomeres during continuous contraction [35], which consists largely of the protein myomesin [36]. Myomesin overexpression was observed in both soleus and heart (Tables 3 and 4), consistent with disintegration of the M-line and dissolution of its constituents. Therefore, the disrupted sarcomeric structure observed in the skeletal muscle of C26-bearing mice could accelerate muscle wasting and indeed reflect a common response to catabolic conditions including cancer cachexia.

Consistent with these morphological changes to myocyte architecture, our proteomics experiments revealed upregulated soluble levels of a variety of muscle fibre proteins, consistent with disintegration of the sarcomere in the C26 mice. These included a variety of myosins (Myh4, Myh7, Mylpf, Myl12, MybpH, Myh2, MybpC2, Myl2), troponins (Tnnt1, Tnni2, Tnnc2), tropomyosins (Tpm2), actins (Acta1, Actn2, Actn3), titin (Ttn), filamin (flnc) and myomesin (MyoM2). The emerging picture is of disintegration of the sarcomere along with loss of its normal connections to the cytoskeleton and organelles such as the mitochondrion. In particular, dissolution of Z-disc and M-line proteins, titin, α-actinin, nebulin and myomesin is likely to be particularly disruptive to sarcomeric integrity.

### Downregulation of energy, metabolism and mitochondrial function in cachexia

Morphological alterations were evident in the mitochondria of cachectic heart muscle (Figure 4C), that could be related to disruption of energy homeostasis in skeletal muscle, and is consistent with our previous observations on cachectic skeletal muscle [24]. These changes included electron–lucent areas which corresponded to a loss of cristae in these mitochondria; suggesting defective oxidative phosphorylation for generating ATP. In addition, swollen mitochondria, which are reported to be associated with cellular ATP depletion, were present [37]. Vesicle-like structures, likely to be autophagic bodies were also observed in some mitochondria which suggested eventual mitochondrial loss via autophagy in cachectic muscle [23]. Therefore, it appears that during cancer cachexia, not only is the sarcomeric structure disrupted, but muscle energy homeostasis may also be disturbed. Such an ultrastructural phenotype may partly explain the reduced grip strength reported in the C26 model [38].

The energy metabolism protein group is almost consistently downregulated across all three muscle types, explaining the lack of energy/strength and fatigue which are hallmarks of cachexia (Tables 2, 3, 4 and Figure 4D). Differentially expressed proteins are representative of multiple pathways, including glycogen metabolism, glycolysis, fatty acid metabolism, TCA cycle, and the electron transport chain. Furthermore myoglobin, a major oxygen reserve in muscle is significantly downregulated in soleus muscle, indicating a further level of compromise of the electron transport chain. The largest number of proteomic changes are focused on the various mitochondrial pathways, with all three muscle types represented (Figure 4D). However, the fast twitch gastrocnemius muscle also has differentially expressed proteins in the glycogen metabolism, glycolysis and fatty acid transport pathways, and therefore appears to be affected across the largest number of metabolic pathways, including glycolysis, glycogen metabolism, TCA cycle, electron transport chain and fatty acid transport/metabolism (Figure 4D). Overall, downregulation of the majority of energy/metabolism proteins not-withstanding several proteins in this group, are upregulated in heart muscle, including Ag1, Pygm, Tpi1 and Uqcrq (Table 4 and Figure 4D), suggesting an attempt to activate homeostatic mechanisms to protect this vital organ.

A multifunctional protein which may be related to energy insufficiency in cancer cachexia is Nme2, which is involved in the synthesis of nucleoside triphosphates other than ATP, and is downregulated in both gastrocnemius and cardiac muscles (Tables 2 and 4). Nucleoside triphosphate kinases are believed to provide GTP to dynamins, which in turn regulate membrane processes such as vesical formation, fusion to other organelles and mitochondrial inner membrane fusion, and knockdown of nucleoside triphosphate kinases resulting in mitochondrial fragmentation [39]. Furthermore, Nme2 also negatively regulates Rho activity, which has a role in cytoskeletal dynamics [40].

### Regulatory proteins and connectivity of the sarcomere to subcellular structures

Given that this is a mouse model of cancer cachexia, it is interesting that the muscle function group of proteins is upregulated in all three muscle types. This effect is a likely result of muscle catabolic processes weakening the muscle fibre structure and solubilising muscle fibre proteins, which would normally be fixed within the sarcomere. Consistent with this possibility, the intermediate filament protein desmin, which regulates sarcomere architecture, and links it to mitochondria and the cell nucleus, is differentially expressed in both gastrocnemius and soleus, being downregulated and upregulated respectively. Desmin-null mice have perturbed muscle architecture, involving both myofibrils and mitochondria, and develop hypertrophic cardiomyopathy [41, 42]. Desmin was also downregulated in cardiac muscle in both biological replicates (Supplementary Materials; Table 3), however did not quite achieve the *p* = 0.05 statistical significance level. Disintegration of the sarcomere during cachexia may be further compounded by desmin dysregulation, and thereby loss of connectivity to mitochondria. Plectin is another protein which associates with the periphery of Z-discs and promotes mechanical integrity of myocytes by connectivity of the myofibril to the cytoskeleton and mitochondria [43–45]. Plectin was upregulated in soleus (Table 3) consistent with loss of sarcomeric integrity. Other proteins within our differentially expressed proteins lists, which may be involved with cytoskeletal assembly/integrity include Pdlim5 [46] (upregulated in gastrocnemius) and Ldb3 (also called ZASP, upregulated in soleus), which interacts with α-actinin and mutations of which are associated with myopathy [47].

### Proteomics and pathology triggers in cancer cachexia

One of the limitations of this case-control study is that it is not clear what the upstream triggers or processes that initiate cancer related muscle wasting are. Additional work, such as a longitudinal study of cancer cachexia progression will be required to determine what these may be. However, there is evidence in our data of several mechanisms which could impact both sarcomere structure and the cellular energy metabolism pathways; (1) *protein folding machinery* may not be up to the demand placed on it. In the soleus, two heat shock proteins (Hsp90aa1 and Hspa8) and the sarcoplasmic/endoplasmic reticulum calcium ATPase (Atp2a1) are all upregulated, suggesting the possibility that the ER may be under stress. Furthermore, several proteins which transport ATP/ADP, Ca^2+^ and inorganic phosphate (Slc25a4, Vdac1 and Slc25a3, respectively) are downregulated, possibly further exacerbating any ER stress, with potentially wide-ranging effects on cellular function, (2) several *inflammation related proteins* (acute phase) are differentially expressed across muscle types, but especially in cardiac muscle. These include; Ahsg (fetuin-A), which is increased in heart and is a protein which is known to increase during inflammation [48], acute phase proteins are upregulated, including fibrinogen chains in soleus (Fga) and heart (Fgg) and α-2-macroglobulin in the heart. Inflammation can be a driver of muscle catabolic processes [49], (3) a variety of *ion or small molecule binding and transport* proteins are differentially expressed. Some of these may be upregulated as an attempt to maintain homeostasis, such as several iron carrier proteins; hemopexin (upregulated in gastrocnemius), serotransferrin (upregulated in heart) and the calcium binding protein parvalbumin (upregulated in heart). However, downregulation of others may drive pathological processes, including the ion channel Vdac1 which is downregulated in both soleus and heart and the inorganic phosphate transporter Slc25a3 which is downregulated in soleus. Four and a half LIM domains protein 1 (FHL1) is upregulated in heart (Table 4). This protein is abundant in skeletal muscle and to a lesser extent heart, and is a multifunctional protein likely to be involved with ion channel binding. Defects in the FHL1 gene are associated with myopathy [50], (4) several proteins in our differentially expressed protein lists may be involved with *DNA binding and transcription regulation*, including the histone proteins Hist2h2bb and Hist2h4 (differentially expressed in both gastrocnemius and soleus muscle), and Nme2 (earlier discussed in the context of GTP synthesis and downregulated in both gastrocnemius and cardiac muscles) is a transcriptional activator of the MYC gene and can also bind DNA non-specifically [51, 52].

### Limitations

A limitation of the current study is the absence of functional analysis which could provide a mechanistic link between cachexia related protein dysregulation and hypothesised metabolic changes associated with cancer progression. However, it does provide a basis/rationale for future work into sarcomere and mitochondrial dysfunction in cachexia. A number of reviews speculating on the molecular mechanisms of muscle wasting in cancer cachexia appear in the literature [34, 49, 53–61] indicating the importance of improving knowledge in this area. Furthermore, studies in the field addressing functional issues in the context of cancer cachexia are increasing [31, 62, 63]. We suggest that discovery based studies such as genomics and proteomics (*i.e*., functional genomics) may not only support/refute current hypotheses, but may also be drivers of new hypotheses. The advantages of “omics” and systems biology approaches have recently been reviewed by Gallagher et al. [60]. Such studies are not only complementary to, but also important precursors to functional studies and are beginning to provide important insights into the molecular drivers of cancer cachexia [16, 17]. We expect that our current study will provide the groundwork for future mechanistic investigations.

## MATERIALS AND METHODS

### Establishment of the colon 26 (C26) carcinoma mouse model of cancer cachexia

The C26 mouse model was established and managed as previously described [13, 24]. Briefly, male Balb/c-DBAj hybrid mice (10–11 weeks old) were obtained from the Animal Resources Centre (Perth, WA, Australia) and housed at the Molecular Physiology Unit (ANZAC Research Institute, Concord, NSW, Australia). Mice were maintained at a constant temperature (22°C) on a 12 hr light: 12 hr dark cycle. Standard rodent chow and water *ad libitum* were freely accessible. All animal work prior to culling was conducted under ethics approval (Sydney South West Area Health Service Animal Welfare Committee; protocol number 2007/006) and has therefore been performed in accordance with the ethical standards laid down in the 1964 Declaration of Helsinki and its later amendments.

For tumour inoculation, 1 × 10^6^ C26 cells in 100 μl RPMI/PS were subcutaneously inoculated in the upper right flank of the Balb/c-DBAj mice (day 0). Non-tumour-bearing (NTB) control mice were injected with 100 μl RPMI medium/PS. Mice were physically restrained by hand holding for the inoculation process, and total body weight of animals was monitored daily. Mice were sacrificed by cervical dislocation at 2 pm to minimise diurnal variation. For the identification of cachexia-specific effects (single endpoint), the experimental endpoint for cachexia was selected so that percentage weight loss in C26-bearing mice ranged from 13–18%, which was within the ethical limit of 20%. Mice were culled on days 14 and 16 post-tumour inoculation to account for the inter-individual variability of weight loss. Lower hindlimbs were selected for study due to enrichment in both slow and fast muscles which could be used for comparison of changes across muscle types in cancer cachexia. Individual muscles soleus and gastrocnemius skeletal muscles and heart were harvested, snap-frozen in liquid nitrogen and stored at −80°C.

### Electron microscopy (EM)

The electron microscopy procedures have previously been described [24]. In brief, muscle tissue was fixed in 2% PFA/2.5% glutaraldehyde in 0.1M sodium cacodylate buffer straight after excision (*n* = 3 for both NTB and C26 groups). Prior to post-fixation in osmium tetroxide/0.2M sodium cacodylate buffer, 2 washes of 1 hr each were done in 0.1M sodium cacodylate buffer. Samples were then progressively dehydrated in 70%, 90% and 100% ethanol, before an overnight incubation in LR white resin (London Resin Company, London, UK). LR white resin was replaced the next day for another hr of infiltration. Samples were then embedded in closed gelatine capsules with fresh resin and polymerised at 60°C for 24 hrs. Ultrathin sections (70 nm) were cut from fixed muscle and stained with 2% uranyl acetate (aqueous) and 2% lead citrate and examined under a Jeol1400 transmission electron microscope (Electron Microscopic Unit, UNSW, NSW, Australia) at a voltage of 100 kV.

### Protein extraction

Muscle extracts were prepared by homogenising muscles in ice-cold buffer [0.15% SDS, 1 mM EDTA, 1 mM EGTA, 1 mM sodium orthovanadate (Na_3_VO_4_), 10 mM sodium fluoride (NaF), 15 mM sodium chloride (NaCl), 20 mM HEPES, 10 mM PMSF, pH 7.5] supplemented with 10 mM PMSF and 1 complete™, EDTA-free protease inhibitor cocktail tablet (Roche Diagnostics, Sydney, Australia) using a Pro200 homogeniser (PRO Scientific Inc, Sydney, Australia). Samples were incubated on ice for 1 hr and supernatant lysates were collected following centrifugation at 14,000g for 10 min at 4°C. Protein samples were divided into aliquots and kept at –80°C before subsequent experimental procedures.

### iTRAQ labeling and 2-D LC-MS/MS

For quantitative MS, iTRAQ isobaric labelling was used (Applied Biosystems, Foster City, CA, USA). iTRAQ labelling, 2-D LC-MS/MS and data analysis was performed as described previously [64, 65]. In brief, protein concentration was determined, and protein (100 µg) from skeletal muscle (gastrocnemius and soleus) or cardiac muscle (whole heart) extracts was precipitated with acetone containing 0.1% v/v HCl at –20°C, centrifuged at 15 000 g, air dried and resuspended in a final concentration of 50 mM NaHCO_3_/0.1% SDS (pH 8.8). For subsequent steps, including trypsinisation, the iTRAQ reagents protocol (Applied Biosystems) was followed, except that 200 mM iodoacetamide (Pierce, Sydney, Australia) was used to alkylate cysteines, and immediately prior to adding iTRAQ reagents, 2.5 µL of 500 mM Na_2_CO_3_ was added to ensure a basic pH 8–9.

Labelled peptides were passed through a strong cation exchange column (SCX), dried under vacuum and passed through a C18 column to desalt the sample (Peptide MacroTrap, Michrom Bioresources, Auburn, CA, USA), eluting with 500 µL CH_3_CN : water : formic acid (50:50:0.1, v:v:v), followed by 200 µL CH_3_CN. To ensure that any peptides present in the SCX flow through would also be captured, an Oasis cartridge (Waters, Sydney, Australia) was used to desalt the SCX flow through component, and the eluent from this step was pooled with the sample from the final C18 step. For LCMSMS analysis the extracted iTRAQ labelled peptides (5 µg) were captured onto a strong cation exchange micro column (0.75 x ∼20 mm, Poros S10, Applied Biosystems) and each of the ammonium acetate eluent steps (12 in total) was captured and desalted on a C18 precolumn cartridge (Michrom Bioresources). After a 10 min wash the pre-column was switched (Switchos, Amsterdam, The Netherlands) into line with an analytical column containing C18 reverse phase packing material (Magic, 5 µm, 200Å, Michrom Bioresources) [66]. Peptides were eluted using a 45 min linear gradient of buffer A (H_2_O:CH_3_CN of 98:2 containing 0.1% formic acid-buffer) to buffer B (H_2_O:CH_3_CN of 20:80 containing 0.1% formic acid-buffer) at 300 nL/min. High voltage (2300 V) was applied through a low volume tee (Upchurch Scientific, Oak Harbor, WA, USA) at the column inlet and the outlet positioned 1 cm from the orifice of an API QStar Pulsar i hybrid tandem mass spectrometer (Applied Biosystems). Positive ions were generated by electrospray and the QStar operated in information- dependent acquisition mode. A Tof MS survey scan was acquired (*m/z* 350–1700, 0.75 s) and the three largest multiply charged ions (counts 420, charge state z2 to z4) sequentially selected by Q1 for MS/MS analysis. Nitrogen was used as collision gas and an optimum collision energy automatically chosen (based on charge state and mass). Tandem mass spectra were accumulated for up to 2.5 s (*m/z* 65–2000) with two repeats.

### Quantitative proteomics data analysis

All of the data was collected using two 8-plex iTRAQ experiments and each run was performed in duplicate (two technical replicates). Automated inline 2-D LC-MS/MS was carried out using 5 biological replicates (*i.e*., 5 control mice and 5 cachexia mice, so 10 mice in total) for gastrocnemius muscle, 2 biological replicates (*i.e*., 2 control mice and 2 cachexia mice so 4 mice in total) for cardiac muscle and a single biological sample for soleus muscle (*i.e*., 1 control mouse and 1 cachexia mouse, so 2 mice in total, as a skeletal muscle comparison tissue). Combined technical replicates were processed using Protein Pilot™ 5.0 software (Applied Biosystems/MDS Sciex), using the Paragon™ Algorithm and selecting thorough ID search effort and biological modifications selected in ID focus. The database used was NCBInr (mouse data current at 20–10–15). ProteinPilot identification and quantitation parameters were set as follows: A detect protein threshold cutoff > 1.3 (unused score) was used, representing a confidence of > 95% in correct protein identification and autobias correction applied to the reporter ion ratios. The iTRAQ reporter labels were applied to the samples as follows: Experiment 1 gastrocnemius C26-bearing mice/NTB control reporter ion ratios were 118/115, 119/116 and 121/117 (gastrocnemius biological replicates 1, 2 and 3); soleus C26-bearing mice/NTB control reporter ion ratio was 114/113; Experiment 2 cardiac C26-bearing mice/NTB control reporter ion ratios were 115/113, 116/114 (cardiac biological replicates 1 and 2) and gastrocnemius C26-bearing mice/NTB control reporter ion ratios were 119/117 and 121/118 (gastrocnemius biological replicates 4 and 5). The background correction option was not used, however autobias correction values were as follows for each of the reporter ion ratios: Experiment 1 gastrocnemius 118/115 (0.8851), 119:116 (0.7827), 121:117 (0.8455), soleus 114/113 (0.5979); Experiment 2 cardiac muscle 115:113 (0.4845), 116:114 (0.7605), gastrocnemius 119/117 (1.0335), 121:118 (0.9808).

### Bioinformatics analysis; STRING v10 and ingenuity pathway

STRING v10 [67] was used to perform network analysis and enrichment of protein groups on the proteins which were found to be differentially expressed in each of the muscle types. For this analysis the default settings were used, the MCL clustering tool was set to a value of 2 and the confidence view selected for output presentation. The protein terms used for each analysis are shown in Tables 2, 3 and 4. STRING v10 was also used to explore enrichment of biological processes, molecular function, cellular components and KEGG pathways against the mouse genome background (only FDR and/or Bonferroni corrected significant values are shown). Ingenuity Pathway Analysis (IPA) software (Qiagen 2015) was used to compare the level of overlap of protein lists between the three muscle types and to explore biochemical networks, diseases and functions which were significantly related to the differentially expressed proteins.

### Proteomics terminology

Use of the terms “up-regulated”, “down-regulated”, and “differential expression level”, are widespread in the proteomics literature and are a convention adopted from the genomics field. Consequently they are sometimes taken to imply genetic regulation. However, in the proteomics context they can only ever represent protein level/s relative to a reference or control sample. Such levels may change due to transcriptional, translational, post-translational or disease mechanisms, and proteomics methods alone cannot establish the mechanism/s by which protein levels change. To help clarify our current usage of this terminology and to avoid false inferences, our use of these terms in the current context only indicates protein levels in the cachexia samples relative to the control samples. Any mechanistic inferences that we make are based on a combination of some or all of our proteomic data, muscle ultrastructural observations and our previously published genomics data.

## CONCLUSIONS

Complementary findings from our morphological and proteomic analyses provide a global picture of the molecular events that underlie skeletal muscle wasting during cancer cachexia; disintegrated sarcomeric structure, increased soluble protein expression of Z-disc components, and disruption of the M-line with increased expression of soluble myomesin. This model for muscle breakdown, triggered by disruption of the Z-line which releases myofibrillar proteins, presents the Z-disc as the most likely initial and rate-limiting step of muscle breakdown in cancer-induced muscle wasting. These processes are further exacerbated by disrupted energy homeostasis, which is another recurrent feature of the pathophysiology of cancer-induced muscle cachexia. Altered mitochondrial morphology, in combination with the reduced expression of proteins regulating substrate and energy metabolism, suggest that muscle cells are likely to be undergoing a state of energy crisis which ultimately results in cancer-induced cachexia. A wide range of biological processes could potentially be affected by the lack of cellular fuel, including muscle contraction, protein turnover and substrate metabolism. However, based on the results presented, it is still unclear if the observed disrupted energy metabolism during cancer cachexia is a consequence or a cause of muscle wasting. The data generated by this iTRAQ proteomics study shows that the genomic changes which we have previously identified are indeed translated into multiple functional downstream changes, which in turn account for muscle wasting in cancer-induced cachexia. Future work should aim to elucidate what the primary triggers are for these changes, and to this purpose, longitudinal studies may be the way forward.

## SUPPLEMENTARY MATERIALS TABLES










