# Supplementary material for: Proteomic profiling of skeletal and cardiac muscle in cancer cachexia: alterations in sarcomeric and mitochondrial protein expression
Source: Oncotarget. 2018 Apr 24;9(31):22001–22. doi: 10.18632/oncotarget.25146 (PMC5955146; doi:10.18632/oncotarget.25146)
Supplement: Supplementary file 1 [file oncotarget-09-22001-s001.pdf]

Proteomic profiling of skeletal and cardiac muscle in cancer cachexia: alterations in sarcomeric and mitochondrial protein expression

SUPPLEMENTARY MATERIALS

Supplementary Table 1: Gastrocnemius muscle full iTRAQ list. See_Supplementary_Table 1

Supplementary Table 2: Soleus muscle full iTRAQ list. See_Supplementary_Table 2

Supplementary Table 3: Heart muscle full iTRAQ list. See_Supplementary_Table 3

Supplementary Table 4: STRING v10 cellular component enrichment of iTRAQ significantly differentially expressed protein lists for gastrocnemius, soleus and heart muscles. See_ Supplementary_Table 4
